# Intervention to improve social and family support for caregivers of dependent patients: ICIAS study protocol

**DOI:** 10.1186/1471-2296-15-53

**Published:** 2014-03-25

**Authors:** Magdalena Rosell-Murphy, Josep Mª Bonet-Simó, Esther Baena, Gemma Prieto, Eva Bellerino, Francesc Solé, Montserrat Rubio, Ilona Krier, Pascuala Torres, Sonia Mimoso

**Affiliations:** 1 Primary Care Team, Serraparera, Institut Català de la Salut. Cerdanyola del Vallès, Barcelona 08290, Spain; 2 Primary Care Unit, Cerdanyola-Ripollet, Institut Català de la Salut. Cerdanyola del Vallès, Barcelona, Spain; 3 Primary Care Agency, Avila, Spain; 4 Primary Care Team, Ripollet, Institut Català de la Salut, Barcelona, Spain; 5 Primary Care Team, Badia del Vallès, Institut Català de la Salut, Barcelona, Spain; 6 Primary Care Team, Canaletes, Institut Català de la Salut. Cerdanyola del Vallès, Barcelona, Spain

**Keywords:** Caregiver burden, Social support, Primary health care

## Abstract

**Background:**

Despite the existence of formal professional support services, informal support (mainly family members) continues to be the main source of eldercare, especially for those who are dependent or disabled. Professionals on the primary health care are the ideal choice to educate, provide psychological support, and help to mobilize social resources available to the informal caregiver.

Controversy remains concerning the efficiency of multiple interventions, taking a holistic approach to both the patient and caregiver, and optimum utilization of the available community resources. .For this reason our goal is to assess whether an intervention designed to improve the social support for caregivers effectively decreases caregivers burden and improves their quality of life.

**Methods/design:**

Design: Controlled, multicentre, community intervention trial, with patients and their caregivers randomized to the intervention or control group according to their assigned Primary Health Care Team (PHCT).

Study area: Primary Health Care network (9 PHCTs).

Study participants: Primary informal caregivers of patients receiving home health care from participating PHCTs.

Sample: Required sample size is 282 caregivers (141 from PHCTs randomized to the intervention group and 141 from PHCTs randomized to the control group.

Intervention: a) PHCT professionals: standardized training to implement caregivers intervention. b) Caregivers: 1 individualized counselling session, 1 family session, and 4 educational group sessions conducted by participating PHCT professionals; in addition to usual home health care visits, periodic telephone follow-up contact and unlimited telephone support.

Control: Caregivers and dependent patients: usual home health care, consisting of bimonthly scheduled visits, follow-up as needed, and additional attention upon request.

Data analysis

Dependent variables: Caregiver burden (short-form Zarit test), caregivers’ social support (Medical Outcomes Study), and caregivers’ reported quality of life (SF-12)

Independent variables: a) Caregiver: sociodemographic data, Goldberg Scale, Apgar family questionnaire, Holmes and Rahe Psychosocial Stress Scale, number of chronic diseases. b) Dependent patient: sociodemographic data, level of dependency (Barthel Index), cognitive impairment (Pfeiffer test).

**Discussion:**

If the intervention intended to improve social and family support is effective in reducing the burden on primary informal caregivers of dependent patients, this model can be readily applied throughout usual PHCT clinical practice.

**Trial registration:**

Clinical trials registrar: NCT02065427

## Background

Longer life expectancy not only increases the total number of elderly individuals but also raises the level of dependency in this age group [1]. Dependency leads to a physical decline that brings with it a need for psychosocial and health care services, coinciding with reduced income in retirement and a lack of adaptability to new situations that put the elderly person at a disadvantage in our rapidly changing world [2].

Despite the existence of formal professional support services, informal support continues to be the main source of eldercare, especially for those who are dependent or disabled [2,3]. Informal support includes the care and services provided by individuals, agencies, and networks other than formal services for the elderly who have some degree of psychophysical dependence; informal caregivers assist with basic and functional needs of daily living during most of the day, without economic compensation for this task [4].

Family members are the main source of informal support for the dependent person, and their support is a clear predictor of the patient’s ability to remain in the home community and delay --or avoid-- institutionalization. The person who takes the major responsibility for this care is defined as the primary caregiver for the dependent patient [5,6].

A key concern for the primary caregiver is the excessive stress defined as caregiver burden [1-3], which has both objective and subjective components. The objective components are the demands, in the broadest sense of the term, to which the caregiver is exposed because of caring for the dependent person. The subjective components include the way the caregiver perceives the caregiving tasks, and specifically his or her emotional response to the experience of caring for a family member. When the emotional involvement is very intense, frequent, or long-lasting, the caregiver’s health and behaviour may be affected [7,8]. The evaluation of psychological well-being, a central component of health-related quality of life (HRQL), is also considered important [9]. Some studies have suggested that this indicator can be improved even when caregivers are excessively stressed, and that research on the health and well-being of caregivers must be complemented with assessments of quality of life or related aspects [10].

A study of informal caregivers in our primary care context [11] assessed the existence and causes of caregiver burden, along with the consequences for the patients, and observed that the overwhelmed caregiver has poor social support, cares for a severely dependent patient, and has been filling this role for a long time. In that study, 66.5% of caregiver burden was due to insufficient social support and variables related to the dependent patient, including age, cognitive status, and degree of dependency.

The adequacy of social support that the caregiver receives is related with the feeling of burden [12]. An inverse relationship has been reported between scores on the Medical Outcomes Study (MOS) test [13,14] which evaluates the social support received, and the Zarit test, which measures caregiver burden. Of all the factors related to caregiver burden, the easiest to modify is social support. Reinforcing social support reduces caregiver burden and improves quality of life. Therefore, an appropriate intervention in this dimension could help the caregiver and other family members, providing tools to confront the changes and respond more effectively to crises resulting from the deterioration of the dependent patient’s status.

In order to sustain the activities of informal caregivers, it is important to attempt to limit or decrease their stress, strengthening their psychological well-being to improve their quality of life. Structured external interventions can improve the support available to informal caregivers, according to numerous recent studies focussed on caregivers for dependent patients in a home health care programme [8] with cerebrovascular accident [15,16] dementia [17-24], and schizophrenia [25].

Professionals on the primary health care team (PHCT) are the ideal choice to educate, provide psychological support, and help to mobilize social resources available to the informal caregiver. The PHCT doctor or nurse has a long-term relationship with the patient that also allows familiarity with the caregiver and care context and the opportunity to offer individualized intervention according to individual needs [26,27].

Various studies with different interventions designed to reduce or prevent caregiver exhaustion have been described [1]. Interventions focussed only on information, support groups, or management of behavioural disorders have not proven their effectiveness. Psychosocial interventions that address multiple dimensions (information about the disease, organization of care needs, practical advice, skill-building for care management, decision-making) are most indicated. Finally, the most successful models of intervention have involved long-term support for informal caregivers over a period of years. In this regard, other family members are among the caregiver’s most accessible resources for ongoing support over time.

The REACH Project is a multi-intervention study that incorporated individual counselling, self-help groups, family therapy, caregiver training, and technological support; it was effective in improving caregiver health and delaying the institutionalization of the dependent patient [27]. The REACH conclusions establish that the intervention with the greatest possibility of success is structured but also personalized to meet the specific needs of the caregiver [27]. Even so, controversy remains concerning the efficiency of multiple interventions, taking a holistic approach to both the patient and caregiver, and optimum utilization of the available community resources [28].

For these reasons, we designed an intervention directed to the caregiver that takes both an individual and family approach to reduce caregiver burden and can be readily implemented by the patient’s assigned PHCT.

### Hypothesis

A multi-factor intervention involving the primary caregiver of a dependent patient, with the goal of improving his or her social and family support and carried out by the professionals of the patient’s regular PHCT, will decrease the burden and increase quality of life for the primary caregiver.

### Objective

#### Primary objectives

1. To determine whether an intervention carried out by primary health care professionals, focussed on the caregiver, the family group, and a self-help group will improve the social support perceived by the caregiver.

2. To evaluate the effectiveness of the intervention in decreasing the caregiver’s burden.

3. To determine whether the intervention improves the caregiver’s perceived quality of life.

#### Secondary objectives

1. To identify population subgroups in which the intervention is most effective, according to caregivers’ reported burden and quality of life.

2. To identify factors that influence the effectiveness of the intervention.

## Methods/design

### Design

Controlled, multicentre, community intervention trial, with random assignment by PHCT group.

### Setting

Nine primary care centres in two regions of Catalonia (Vallès Occidental and Vallès Oriental), Spain.

### Type of participants

Informal primary caregivers for patients in the home health care program, identified from electronic medical records (eCAP database).

#### Inclusion criteria

Adults (older than 18 years) identified in the eCAP database as the primary caregiver for a dependent patient, and who act as caregivers without remuneration.

Both the caregivers and the dependent patients have an active clinical record in a participating primary care centre.

#### Exclusion criteria

1. Caregivers for intermittent periods, independently of the length of care provided.

2. Caregivers who have provided less than one year of ongoing care.

3. Caregivers with any communication problem (psychiatric disorders, etc.) that makes them difficult to interview.

### Study sample

#### Size

To avoid contamination of the intervention, randomization was done at the PHCT level. In a descriptive study of 500 primary caregivers throughout Catalonia, we obtained a standard deviation (SD) of 7.32 on the Zarit short test, or a difference of 4 points between tests at the beginning and end of the intervention. For a simple random design, accepting an alpha risk of 0.05 and a beta risk of 0.20 in a two-tailed test and 0.2 loss to follow-up, the sample must include 128 participants, 64 in the intervention group and 64 controls. Considering an intraclass correlation coefficient of 0.05 and an average of 25 informal primary caregivers of ATDOM patients per PHCT, the design effect is 2.2; therefore, a total of 282 caregivers are required for the study (141 per group). Sample size was calculated using Epidat 3.1 software.

### Measurement of main outcome variables

#### Dependent variables

##### Social support

The 20-item MOS test [13,14], which contains one question about the social network and 19 items assessing four dimensions of social support, measures the caregiver’s subjective perceptions of the amount and types of social support received. Responses use a five-point Likert scale, with an overall range of 19 to 95 points; below the cut-off of 57, the respondent’s social support is considered insufficient [14].

##### Caregiver burden

The short-form Zarit test [29] assesses this variable, using the cut-off score of 17 to determine the existence of burden, based on a published pilot study [30].

##### Caregiver perception of quality of life

The Short-Form 12 (SF-12) health questionnaire [31,32] elicits information in two areas, physical and mental health, and yields a profile of the respondent’s health status. The 12 responses are scored on a Likert scale. To interpret the score, values are standardized to population norms, so that 50 (SD 10) is the average for the general population. Lower values are considered worse than the reference population [33].

### Measurement of secondary variables and effect modifiers

#### Independent variables

a) The Caregiver:

• Sociodemographic data: age, sex, marital status, employment status, educational level

• Time in caregiver role (in months)

• Score on Goldberg anxiety and depression scales [34,35].

• Family data:

Family structure: World Health Organization classification, depending on household composition [36].

Phase of family life cycle (World Health Organization classification, modified by De la Revilla [37]): *Phase 1*, Formation (from marriage until the birth of the first child); *Phase 2A*, Expansion from first child’s birth to 11 years of age; *Phase 2B*, Expansion from the first child’s 11th birthday to the birth of the last child; *Phase 3*, End of the Expansion (from the birth of the last child until the first child leaves home); *Phase 4*, Contraction (from the first child’s departure until the last child leaves home); *Phase 5*, End of Contraction (from the last child’s departure until the first spouse’s death; and *Phase 6*, Dissolution (from the death of the first spouse until the other spouse dies).

Social network: Defined as the people who contribute support or assistance to an individual or family, and taken from the response to the first question on the MOS test, which specifically addresses this question.

Family function: The perceived functionality of the family unit, assessed using the Apgar family questionnaire [38], which has 5 questions with 3 possible responses. Each answer scores between 0 and 2 points, for a range of 0 to 10. A total score of 7 or more points indicates a functional family and a lower score suggests family dysfunction.

• Stressful life events: The Holmes and Rahe Social Readjustment Rating Scale [39,40] has 43 items that measure stressful life events in “life-change units” over the previous 12 months. Respondents scoring more than 150 life-change units are considered to be at risk.

b) The dependent patient

Sociodemographic data: age, sex, marital status, educational level. Degree of dependence (Barthel Index) for activities of daily living [41] at the time of study inclusion.

Cognitive status: Pfeiffer Test, [42,43] consisting of 10 true-or-false questions. Between 0 and 2 errors is considered normal intellectual function, 3 to 7 errors indicates potential deterioration, and 8 to 10 errors a severe intellectual deficit.

Reason for inclusion in home health care programme [44]: Eligible patients are classified into target groups according to different minimum common criteria, published by the Catalan government’s Department of Health and Social Security in 1996:a) *Chronic disease criterion*: persons affected by chronic, neurologic, respiratory, rheumatologic or other processes; b)*Terminal conditions criterion*: persons in terminal stage of neoplasm, AIDS, or geriatric health conditions; c) *Cognitive deterioration criterion*: persons with a permanent cognitive deficit; d)*At*-*risk criterion*: persons included in health promotion and disease prevention programmes because of social isolation, advanced age, physical or psychosocial dependency, architectural or other barriers. Visual analogue scale (0 to 100) for emotional status Subjective health assessment (0 to 100 points).

### Conduct of the study

From electronic medical records, a list was obtained of the individuals identified as the primary caregiver for patients receiving home health care services. A simple random sample was selected. The corresponding doctor and/or nurse contacted the potential participants by telephone to explain the study and ask if they would be interested in participating. Interested caregivers were visited at home by an independent interviewer, who provided an information sheet about the study and requested signature of informed consent to participate. To avoid any potential bias, the interviewer was simple-blinded to the participant’s group assignment. An administrator compiled the baseline data for all participants into a database for analysis.

Once the 282 study participants were identified, they were randomly assigned by PHCT to the intervention or control group. All PHCTs randomized to implement the intervention received the following standardized training:

1. Seminar on neurolinguistic approaches that facilitate communication between health professionals and caregivers, helping the health care team improve their skills related to active listening and recognition of unexpressed needs.

2. Workshop on approaches to family care, providing tools needed to understand and work with the family life cycle, social support structures, and genograms.

3. Working session on family interviews and understanding family relationships and their implications for caregiving, with specific sessions on strategies that foster collaboration and synergy between family members.

4. Training on working with groups, conducted by primary care professionals with appropriate training and experience.

5. Practical workshops designed to standardize the intervention strategies to be used by all participating PHCTs.

### Caregiver intervention

The intervention was designed to activate and strengthen the social support for each caregiver. There are four dimensions to social support: instrumental support, positive social interaction, informational/confidential support, and emotional support. Previous intervention studies have observed that differentiated approaches are needed to achieve improvements in knowledge, attitude, and quality of life. This led us to design a multifactorial intervention [45,46], as follows:

a) *Individualized intervention* (*90 minutes*): One counselling session with each caregiver, providing information about formal public and private resources that are available, according to the needs in each case, including relevant materials and emotional support (instrumental/emotional dimensions). In these sessions, the caregiver’s main concerns and problems in properly carrying out his or her care tasks are identified and prioritized. The individuals to be invited to the family intervention session are also identified.

b) *Family Intervention* (*1*–*2 group sessions*, *90 minutes each*): Sessions address the informational/confidential, emotional, and positive social interaction dimensions.

c) *Group educational sessions* (*up to four 60*-*minute sessions*, *depending on caregiver availability*): Informational/confidential, emotional and positive social interaction dimensions are addressed in three-part sessions: informational presentations on theory, time for sharing and discussion, and a relaxation exercise. Individuals learn from their own experiences and cognitive models, and make changes based on new information and the experiences of others.

### Content outline

• **Session 1**:

• Introduction. Objectives of the intervention

• Health education concerning the patients’ disease processes, with the groups organized around similar diseases if possible.

• Sharing and discussion, taking advantage of the content of participant comments to prepare following sessions, offer answers to questions that arise, and provide emotional support.

• Relaxation exercise

• **Session 2**:

• Introduction. Objectives of the intervention

• Health education concerning aspects of self-care for the caregiver (e.g., posture, nutrition, rest), information from the social worker about available resources, and response to questions raised in Session 1.

• Sharing and discussion, taking advantage of the content of participant comments to prepare following sessions, offer answers to questions that arise, and provide emotional support.

• Relaxation exercise

• **Session 3**:

• Introduction. Objectives of the intervention

• Health education about additional aspects of self-care, as well as the patient’s hygiene, mobility and nutrition, and response to questions raised in Session 2.

• Sharing and discussion, taking advantage of the content of participant comments to prepare the final group session, offer answers to questions that arise, and provide emotional support.

• Relaxation exercise

• **Session 4**:

• Introduction. Objectives of the intervention

• Health education concerning medications and safety (e.g., avoiding falls and accidents in the home, orthopaedic resources)

• Sharing, discussion, and emotional support

• Relaxation exercise and closing

In addition to monthly telephone follow-up, participants receive a direct telephone number to consult with a health professional for support as needed.

### Control group

Caregivers in the control group received the usual follow-up provided through home health care by their PHCT, which includes scheduled visits to the home and additional specific attention as requested. This is the established means of maintaining a relationship between the doctor, nurse, and caregiver to facilitate patient follow-up and progress.

Following normal practice, needed resources were offered and all activities for caregivers that were being offered prior to the study period continued to be made available.

### Follow-up

When the intervention had been completed, an independent interviewer contacted all caregivers in both groups by telephone to conduct a final survey.

### Analysis strategy

An initial comparability analysis ensured homogeneity between the populations of the intervention and control groups. Descriptive analysis of all variables will include the Student t and Mann–Whitney tests to compare means between two categories, ANOVA to compare three categories, chi-square to compare two categorical variables, and chi-square test for trends when one of the variables is ordered.

To determine which factors can be associated with the intervention, binary or ordinal unconditional logistic regression will be used to assess categorical variables, adjusting for potential confounding factors and clinical variables of possible relevance. The dependent patient’s death or institutionalization is considered an end-point of the study. All analyses will be based on intention to treat. All two-tailed statistical tests will use a 95% confidence level. The software packages SPSS for Windows version 15, STATA and HLM 6 will be used for all analyses.

**Figure 1:**
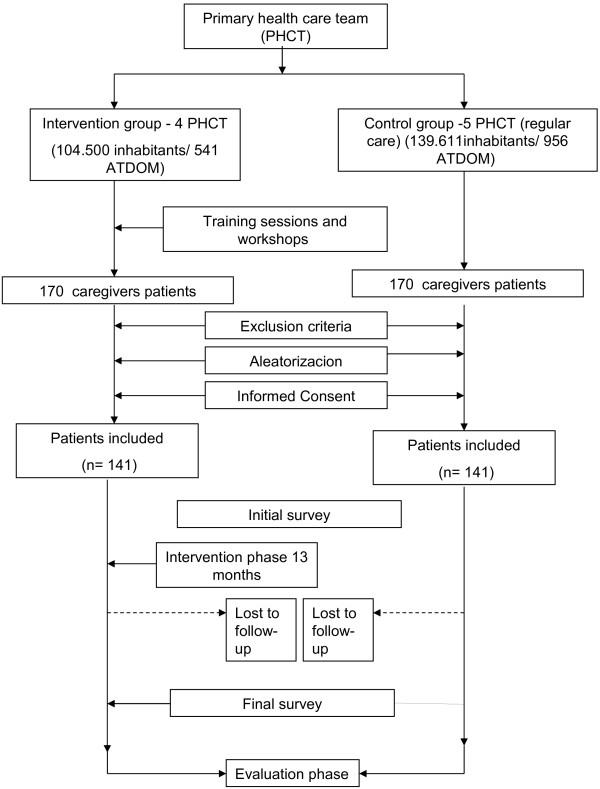
General outline of the study.

The work plan and timeline is detailed in Figure 1.

### Ethical aspects

During the first direct contact with the caregiver, the project was presented together with a written request for voluntary participation. An information sheet provided the objectives and detailed characteristics of the study. A consent form described the general and specific ethical aspects related to the right to privacy, anonymity and confidentiality, to withdraw from the study without any penalty, and to receive study information. Participants were informed that, in order to guarantee the accuracy of study data, permission is required for researchers and health care authorities and/or members of the Ethics Committee for Clinical Research to gain access to their electronic medical records, with strict protection of the confidentiality of personal data. Only participants who provided signed informed consent were included.The protocol fully complies with Spain’s data protection law (15/1999) and recent amendments (Royal decree 1720/2007) and was approved by the IDIAP Ethics Committee for Clinical Research (Comitè Etic d’Investigació Clinica del Institut de Investigació en Atenció Primària Jordi Gol) on March 2, 2009.

#### Limitations

Although 20% loss to follow-up was calculated, the high mortality rates in the dependent population could exceed this estimate. The protocol provided for the addition of replacement caregivers if the initial sample decreased more than 20%.

The caregiver assessments (interviews and tests) were blinded and independent for both groups, carried out by members of the research team not participating in the clinical care of the caregivers. There may be some variation between the participating PHCTs in the implementation of the intervention. Nonetheless, the training and workshops provided to PHCT teams in the intervention group are designed to standardize and contribute to homogeneity in implementing the intervention; usual care in the control group follows guidelines set out by the Catalan Health Service.

## Discussion

The high levels of caregiver burden reported by other studies in our environment [4,5] must sensitize us to the need to study the most appropriate instruments of caregiver support to diminish this burden. Family support decreases as years go by, and a negative first experience with caregiving can mean that the same person will not want to do it again and may prematurely choose the alternative of institutionalizing a dependent family member.

Numerous types of interventions aiming to improve patient care and caregivers self-care have been described, involving caregivers for patients with different chronic diseases and high dependency. Some studies demonstrate the efficacy of these interventions [12,13,15-24], although the results are moderate. All of this increases interest in establishing interventions that significantly improve the health of caregivers and consequently of the patients in their care.

This study applies various intervention strategies, in line with studies that demonstrate better response with combined interventions [45,46]. These include the *individualized* caregiver interview, directed at providing information, educating and modifying cognitive abilities to confront stressors and how they are perceived [45]; *family interview* to address the caregiver’s environment; and *group sessions* for caregivers that focus on affective considerations and the emotional response of the caregiver [46]. Paying attention to the needs expressed by caregivers, together with the interventions proposed, facilitates the goal of improving family and social support.

If the final results obtained in our study show that increasing and strengthening the primary caregiver’s social support improves quality of life and decreases caregiver burden, this will allow us to adopt a new perspective on home health care. A new strategy focussed on the family system and on caring both for the caregiver and dependent patient, providing more comprehensive support and better planning through the primary care system, may strengthen preventive efforts and improve care. The critical situations that occur when caring for dependent patients could also be addressed in ways that are more satisfactory for both the caregiver and patient, and even for the responsible health care professional. An intervention that focuses on procedural and behavioural changes supported by specific training for PHCT personnel will be relatively easy to extend throughout the primary care network.

## Competing interests

The authors declare that they have no competing interests.

## Authors’ contributions

MR, JMB, and GP were responsible for the protocol design, project follow-up, and evaluation. FS, FN, EB, IK, AÁ, SK, MP, MR, EB, SM, and EV were responsible for developing materials for the caregivers. JMB, FS, EB, and IK were responsible for designing the intervention. EB was responsible for contact with the team leaders and coordinating the interviewers. FS, JMB, EB, and IK participated in the training of the key staff members of the PHCTs in the intervention group. PT, SK, MP, NA, DA, MRM, and SM were responsible for training related to group education sessions. All authors read and approved the final manuscript.

## Pre-publication history

The pre-publication history for this paper can be accessed here:

http://www.biomedcentral.com/1471-2296/15/53/prepub
